# Prevalence of low trabecular bone score and its association with disease severity and activity in patients with axial spondyloarthritis

**DOI:** 10.1038/s41598-023-43321-5

**Published:** 2023-09-27

**Authors:** Pannarat Saisirivechakun, Ajanee Mahakkanukrauh, Chatlert Pongchaiyakul, Trirat Boonya-ussadorn, Pongthorn Narongroeknawin, Rattapol Pakchotanon, Paijit Assavatanabodee, Sumapa Chaiamnuay

**Affiliations:** 1 Rheumatic Diseases Unit, Department of Internal Medicine, Phramongkutklao Hospital and College of Medicine, 315 Ratchawithi Road, Ratchathewi District, Bangkok, 10400 Thailand; 2 Division of Rheumatology, Department of Medicine, Faculty of Medicine, Khon Kaen University, Khon Kaen, Thailand; 3 Division of Endocrinology and Metabolism, Department of Medicine, Faculty of Medicine, Khon Kaen University, Khon Kaen, Thailand; 4 Division of Nuclear Medicine, Department of Radiology, Phramongkutklao Hospital and College of Medicine, Bangkok, Thailand

**Keywords:** Biomarkers, Diseases, Rheumatology

## Abstract

Axial spondyloarthritis (axSpA) increases the risk of osteoporosis and vertebral fractures. Bone mineral density (BMD) measured by dual X-ray absorptiometry (DXA) has limitations in axSpA patients. Trabecular bone score (TBS) indirectly assesses bone microarchitecture and can be used to predict fracture risk. However, few studies have investigated the role of TBS in axSpA patients. The objective of this study were to compare TBS between axSpA patients and 1:1 sex- and age-matched healthy volunteers and determine factors associated with low TBS in axSpA patients. A cross-sectional study was conducted in two tertiary-care hospitals. A total of 137 axSpA patients and healthy volunteers were enrolled. Demographics, disease characteristics, and risk factors for osteoporosis were recorded. TBS, BMD at the lumbar spine, hip, and vertebral fractures were assessed by DXA. Low TBS was defined as a TBS value < 1.230. Factors associated with low TBS were examined by logistic regression. Most patients were male (75.9%) and tested positive for HLA-B27 (88.3%). The mean (SD) age was 42.8 (12.0) years. The mean (SD) of TBS in the axSpA patients was lower than those in the healthy volunteers [1.402 (0.107) vs 1.440 (0.086), respectively; p = 0.002]. The mean (SD) of lumbar BMD in the axSpA patients was higher than in healthy volunteers [1.186 (0.212) vs 1.087 (0.124), p < 0.001], whereas the mean (SD) of femoral neck BMD in the axSpA group was lower than that in the healthy volunteers [0.867 (0.136) vs 0.904 (0.155), p = 0.038]. Disease severity as indicated by sacroiliac joint fusion and a high ASDAS score were associated with low TBS with the odds ratios (95% confidence interval) of 11.8 (1.2–115.4) and 5.2 (1.6–16.9), respectively. In conclusion, axSpA patients had a higher prevalence of low TBS than healthy volunteers. Sacroiliac joint fusion and a high ASDAS score were associated with low TBS.

## Introduction

Axial spondyloarthritis (axSpA) is a chronic inflammatory disease that primarily affects the axial joints (spine and sacroiliac (SI) joints), as well as peripheral joints and entheses. Extraspinal manifestations such as peripheral arthritis, anterior uveitis, psoriasis, colitis, and rare manifestations such as pulmonary fibrosis and amyloidosis are also affected by axSpA^1^. Although the pathogenesis is unknown, several hypotheses have been proposed, including the arthritogenic peptide, misfolding, molecular mimicry, and cell-surface HLA-B27 homodimer. As a result, a variety of cytokines are produced, which play a role in both the inflammatory process and the formation of new bone. During the inflammatory process, those cytokines, namely interleukin-1 (IL-1), IL-6, IL-17/23, and tumor necrosis factor –α (TNF-α), stimulate the production of receptor activator of nuclear factor kappa-B ligand, which is a potential stimulator of osteoclast differentiation, resulting in bone loss in axSpA patients^2,3^. Meanwhile, bone morphogenetic protein and Wnt signaling pathways induce osteoblast differentiation to contribute to new bone formation, leading to ankylosis and disability^3,4^.

Osteoporosis is one of the most common comorbidities in axSpA patients^5^. Paradoxically, new bone formation occurs resulting in ankylosis of the spine and SI joints. The prevalence of osteoporosis in axSpA patients were reported between 11.7 and 34.4%^6,7^. Several studies have found that long-standing forms, spinal ankylosis, high inflammatory markers, alcohol consumption, corticosteroid use, and vitamin D deficiency were risk factors for low bone mineral density (BMD) in axSpA patients^5,6^. Importantly, patients with axSpA are at a higher risk of vertebral fractures (VFs) than the general population^8^. Furthermore, elevated C-Reactive Protein (CRP), disease duration, and hyperkyphosis have also been linked to an increased risk of VFs^5^.

The dual-energy X-ray absorptiometry (DXA) is the gold standard tool to assess bone mineral density (BMD). BMD is calculated by measuring bone mineral content divided by the area of interest. Every one standard deviation (SD) different from a mean healthy young adult is reported as T-score. According to the WHO criteria, osteoporosis is defined as a BMD T-score of −2.5 or less measured by DXA at the spine, hip, or forearm^9^. Furthermore, DXA-BMD at the femoral neck is used in the fracture risk assessment tool FRAX which has been implemented in several guidelines. The 2019 update of the American College of Rheumatology (ACR)/Spondylitis Association of America/Spondyloarthritis Research and Treatment Network Recommendations for the Treatment of Ankylosing Spondylitis and Non-radiographic axSpA suggested that axSpA patients be screened for osteoporosis with DXA^10^. However, the BMD from DXA may inadvertently increase in axSpA patients due to syndesmophyte formation^11^.

In addition to BMD measurement, DXA can assess other factors that are helpful in determining the risk of developing fragility fracture risk, such as bone geometry by hip structural analysis and hip axis length^12^, vertebral fracture assessment (VFA), bone strength by bone strain index (BSI)^13^, and bone microarchitecture by trabecular bone score (TBS).

The TBS evaluates the subtle variations in pixel grey level within the lumbar DXA image, thereby providing an indirect measurement of bone microarchitecture as trabecular number, trabecular separation, and connectivity density without additional radiation exposure to the patients. A high TBS value is indicative of a strong microarchitecture that exhibits resistance against fractures, whereas a low TBS value implies a fragile microarchitecture that is prone to fractures^14^. Several studies have reported that TBS was associated with fracture risk in postmenopausal women, particularly VFs. TBS, unlike DXA, is less likely to be affected by syndesmophytes. Therefore, TBS may be valuable in fracture risk assessment in axSpA patients^14,15^.

The objective of this study was to compare TBS in Thai axSpA patients with age- and sex-matched normal individuals and examine the factors associated with low TBS in axSpA patients.

## Methods

### Setting and subjects

A cross-sectional study was conducted at Phramongkutklao and Khon Kaen University Hospitals (tertiary care settings) between January 2020 and February 2021. Patients aged 18 years and older who fulfilled the ASAS classification criteria for axSpA were consecutively recruited^16^. Patients with a history of cancer or a first-time cancer diagnosis (regardless of stage), chronic kidney disease at least stage IV^17^, hyperthyroidism, hyperparathyroidism, pregnancy, and/or lactation were excluded. Each patient’s healthy volunteer participants were randomly selected from the general Thai population and matched for age and sex with a ratio of 1:1. The Royal Thai Army Medical Department Institutional Board Review and the Khon Kaen Hospital Institutional Review Board in Human Research approved the study with the approved study numbers R178h/62 and HE631141, respectively and confirmed that this research complied with the Declaration of Helsinki. The written informed consent was obtained from all participants and/or their legal guardians prior to entry to the study.

### Bone mineral density and trabecular bone score measurements

BMD was measured at the lumbar spine (LS), femoral neck (FN), and total hip (TH) using GE-Lunar iDXA (#210754) and GE-Lunar DPX Duo densitometer (GE Healthcare, Madison, WI, USA). Osteoporosis was defined as a BMD T-score of -2.5 or less in postmenopausal women and men aged ≥ 50 years. Whereas, a Z-score of -2.0 or less was defined as being below the expected range for age (low BMD) in premenopausal patients and men aged < 50 years^18^. The TBS was obtained from the DXA scan (Medimaps TBS iNsight) at the lumbar spine in both settings. The TBS cut-offs used to categorise TBS groups are obtained from the metanalyses of individual-level data covering 17,809 men and women in 14 prospective population-based cohorts around the world. These groups are categorised as normal TBS, partially degraded (or intermediate TBS), and degraded (or low TBS) when TBS values are above > 1.310, between 1.230 and 1.310, and below < 1.230, respectively^19^. Fractured vertebrae were excluded from lumbar DXA analysis.

### Radiography and vertebral fracture assessment

The vertebral fracture assessment (VFA) by DXA and/or the lateral thoracolumbar X-rays were used to determine the presence of VFs. VFs were defined using Genant’s semiquantitative method^20^ and reviewed by a certified nuclear radiologist. The spinal deformity index (SDI) was calculated by summing the score from 13 vertebrae (T4 to L4). For each vertebra, a visual semiquantitative score of 0, 1, 2, or 3 was assigned for no fracture or mild, moderate, or severe fracture, respectively^21^. In this study, the SI joints were assessed by radiograph. The modified New York criteria were used to classify sacroiliitis^22^.

### Measurements in axial spondyloarthritis patients

Demographic and clinical data including age, gender, body mass index (BMI), history of alcohol and smoking, duration of disease (defined as the time since axSpA diagnosis), family history of axSpA, current medications [i.e., non-steroidal anti-inflammatory drugs (NSAIDs), proton pump inhibitors (PPIs), sulfasalazine, anti-TNF, anti-IL-17, and anti-osteoporotic agents (anti-OP)] were recorded.

In the present study, patient global assessment (PGA), the Bath Ankylosing Spondylitis Disease Activity Index (BASDAI) in Thai version^23^, the Ankylosing Spondylitis Disease Activity Score [ASDAS-CRP or ASDAS-ESR if C-reactive protein (CRP) was not available], the Bath Ankylosing Spondylitis Functional Index (BASFI) in Thai version^23^, and the Bath Ankylosing Spondylitis Metrology Index (BASMI) were used to assess disease activity^24^.

### Statistical analysis

Statistical analysis included computing the percentage frequencies for categorical variables and means (standard deviation, SD) or medians (interquartile ranges, IQR) for continuous variables. Comparisons of categorical variables were made using the chi-squared or Fisher’s exact test, as appropriate. Continuous variables were tested for normality using a Shapiro–Wilk test and were compared using Student’s *t*-test and one-way ANOVA or the Mann–Whitney U test and the Kruskal–Wallis test to compare two and three groups’ means, respectively. Medians with IQR were used for non-normally distributed variables. Univariate and multiple logistic regression analyses were used to determine the relevant risk factors for low TBS. Only risk factors that had univariate associations of *P*-values < 0.20 were further considered in subsequent multivariate models. The lack of collinearity was confirmed by testing variance inflation factors. *P*-values < 0.05 were considered statistically significant. All statistical analyses were performed using SPSS software (IBM SPSS Statistics for Windows, Version 23.0. Armonk, NY: IBM Corp.).

### Ethics approval and consent to participate

The ethical approval was approved by the Institutional Review Board of the Royal Thai Army Medical Department and the Human Research Ethics Committee of Khon Kaen University. The reference numbers are R178h/62 and HE631141, respectively. This research complied with the Declaration of Helsinki. The written informed consent was obtained from all participants and/or their legal guardians prior to entry to the study. 

## Results

As shown in Table 1, the study population recruited 137 axSpA patients (115 from Phramongkutklao Hospital and 22 from Khon Kaen University Hospital) and 137 matched healthy volunteers. The majority of participants were male (75.9%), and 34.3% of the women were post-menopausal. The mean (SD) of age and mean duration of disease were 42.8 (12.0) years and 86.8 (96.9) months, respectively. HLA-B27 was found in 88.3%, whereas the family history of axSpA was found in only 13.1%. Patients with axSpA had concomitant anterior uveitis and psoriasis (5.8% and 1.5%, respectively). In axSpA patients, alcohol consumption who used greater than or equal to 3 units/day was found in 19.7%, whereas patients with smoking including ex-smoker were found in 35.8%.

**Table 1:** Clinical characteristics of healthy volunteers and axial spondyloarthritis patients.

Variable	Healthy volunteers (n = 137)	AxSpA patients (n = 137)	p-value
Age, mean (SD), years	42.80 (12.04)	42.80 (12.04)	1.000
Male, n (%)	104 (75.91)	104 (75.91)	1.000
Body mass index, mean (SD), kg/m^2^	22.79 (3.27)	23.91 (4.95)	0.156
Bone mineral density (BMD)			
Lumbar spine: mean (SD), n = 137	**1.087 (0.124)**	**1.186 (0.212)**	< 0.001*
Femoral neck: mean (SD), n = 128	**0.904 (0.1546)**	**0.867 (0.136)**	0.038*
Total hip: mean (SD), n = 128	**0.948 (0.1470)**	**0.919 (0.160)**	0.119
Low BMD^$^/osteoporosis: any site, n (%)	6 (4.4)	13 (9.6)	0.093
Lumbar spine	5 (3.6)	8 (5.8)	0.394
Femoral neck	3 (2.2)	5 (3.9)	0.421
Total hip	1 (0.7)	7 (5.5)	0.024*
Trabecular bone score (TBS) values, n (%)			
TBS < 1.23	2 (1.46)	10 (7.35)	0.010*
TBS 1.23–1.31	8 (5.84)	16 11.76)	
TBS > 1.31	127 (92.70)	110 (80.88)	
TBS: mean (SD), n = 136	**1.440 (0.086)**	**1.402 (0.107)**	0.002*

*AxSpA* axial spondyloarthritis.

*p < 0.05.

^$^Included individuals with Z-score below −2.0.

Significant values are in bold.

The mean LS-BMD was significantly higher in axSpA patients compared with the healthy volunteers. In contrast, the mean FN-BMD was significantly lower in axSpA patients than in the healthy volunteers. There was no significant difference in TH-BMD between the two groups. There were 13 patients (9.6%) within the axSpA group who met the criteria for osteoporosis or bone mass below the expected range for age. The prevalence of low BMD/osteoporosis at TH was significantly higher in axSpA patients than that in the healthy volunteers, but it was not significantly different between axSpA patients and the healthy volunteers at LS and FN (Table 1). The mean (SD) TBS was significantly lower in patients with axSpA compared to healthy volunteers, with values of 1.402 (0.107) and 1.444 (0.107), respectively (p = 0.002), and exhibiting a statistical power of 94.7%. The computed effect size was 0.391. The prevalence of degraded bone/low TBS in the axSpA patients was also significantly higher than that in the healthy volunteers (7.35% vs 1.46%, p = 0.010). Due to the limited number of patients in the low TBS and osteoporosis group, the clinical significance of these findings might be unclear, thus warranting a cautious interpretation.

As depicted in Table 2, axSpA patients with degraded bone/low TBS group had higher CRP levels, disease activity scores (including PGA, BASDAI, BASFI, BASMI, and ASDAS), and prevalence of grade 4 sacroiliitis and current NSAIDs use than those in normal and high TBS groups. In addition, axSpA patients with degraded bone/low TBS group had a significantly shorter disease duration when compared with other groups. We found that patients with axSpA who had never received anti-TNF or anti-OP medication had lower TBS than patients who had received those medications. (p = 0.037 and p = 0.028, respectively). Furthermore, axSpA patients with degraded bone/low TBS group had significantly lower BMD at LS, FN, and TH than those with intermediate/ normal TBS groups. The radiographic VFs were found in 10 patients (7.5%, 10/134). The prevalence of VFs was significantly higher in patients with degraded bone/low TBS group as compared with patients with intermediate and normal TBS groups (30.0% vs 6.67% vs 5.5%, p = 0.019, respectively).

**Table 2:** Characteristics of patients with axial spondyloarthritis, stratified by trabecular bone score.

Variable	Degraded bone/low TBS (TBS < 1.23, n = 10)	Partially degraded bone/intermediate TBS (TBS = 1.23–1.31, n = 16)	Normal bone/normal TBS (TBS > 1.31, n = 110)	P-value
Age, mean (S.D.), years	38.4 (15.3)	48.7 (15.81)	42.4 (10.9)	0.072
Male, n (%)	6 (60.0)	12 (75.0)	85 (77.3)	0.474
BMI, mean (S.D.), kg/m^2^	23.2 (1.7)	22.9 (4.1)	23.92 (4.9)	0.668
Alcohol intake ≥ 3 unit/day, n (%)	4 (40.0)	2 (12.5)	21 (19.1)	0.209
Ex- or current smoker, n (%)	4 (40.0)	10 (62.5)	35 (31.8)	0.056
Post-menopause, n (%)	1 (8.3)	1 (8.3)	10 (83.3)	0.924
Laboratory findings				
ESR, mean (S.D.), mm/h, n = 126	42.6 (27.2)	30.7 (21.3)	25.8 (23.2)	0.105
CRP, mean (S.D.), mg/l, n = 119	54.3 (29.2)	14.9 (21.7)	15.5 (26.1)	0.001^§,†^
**CRP ≥ 5 (mg/L), n = 119, n (%)**	8 (100)	9 (64.3)	54 (55.7)	0.046^†^
Disease-related data				
Duration of disease, mean (S.D.), months	28.4 (50.2)	116.4 (118.7)	86.7 (94.8)	0.008^§^^,^^†^
PGA, mean (S.D.)	58.0 (23.0)	32.81 (23.8)	32.9 (25.3)	0.011^§^^,^^†^
BASDAI score, mean (S.D.)	4.9 (1.1)	3.2 (2.3)	2.8 (2.2)	0.011^†^
**BASDAI ≥ 4, n (%)**	8 (80)	8 (50)	29 (26.4)	0.001^†^
BASFI score, mean (S.D.)	3.8 (2.2)	2.8 (2.0)	2.2 (2.4)	0.024^†^
BASMI score, mean (S.D.)	5.3 (1.5)	4.8 (2.1)	3.6 (2.2)	0.003^†,¶^
ASDAS, mean (S.D.)	4.1 (0.7)	2.5 (1.1)	2.4 (1.1)	< 0.001^§^^,^^†^
**ASDAS ≥ 2.1, n (%)**	10 (100)	10 (62.5)	62 (56.9)	0.028^†^
Grade 4 sacroiliitis, n (%)	9 (90.0)	9 (56.3)	35 (32.1)	0.001^†^
Presence of syndesmophytes, n (%)	8 (80.0)	12 (75.0)	58 (53.7)	0.095
Medications				
Current glucocorticoid use, n (%)	1 (7.1)	0	13 (92.9)	0.348
Current NSAIDs use, n (%)	10 (100.0)	9 (56.3)	46 (41.8)	0.002^§^^,^^†^
Current PPIs use, n (%)	6 (60.0)	6 (37.5)	38 (34.6)	0.278
Current sulfasalazine use, n (%)	5 (50.0)	10 (62.5)	62 (56.4)	0.816
Current anti-TNF use	0	6 (37.5)	18 (16.36)	0.037^§^^,^^¶^
Current anti-IL-17 use	0	1 (6.3)	1 (0.9)	0.233
Current anti-OP use	0	3 (18.8)	4 (3.6)	0.028^¶^
Bone mineral density (BMD)				
Lumbar spine: mean (SD), n = 136	0.942 (0.156)	1.128 (0.196)	1.211 (0.199)	< 0.001^†^
Femoral neck: mean (SD), n = 128	0.764 (0.768)	0.738 (0.172)	0.897 (0.124)	< 0.001^†,¶^
Total hip: mean (SD), n = 128	0.780 (0.097)	0.795 (0.180)	0.952 (0.152)	< 0.001^†,¶^
Low BMD^$^/osteoporosis				
Lumbar spine	5 (50.0)	2 (12.5)	1 (0.9)	< 0.001*
Femoral neck	2 (20.00)	2 (14.3)	2 (1.9)	0.007^†^^,^^¶^
Total hip	2 (20.00)	2 (14.3)	3 (2.9)	0.022^†^^,^^¶^
Vertebral fractures^#^, n = 134	3 (30.0)	1 (6.7)	6 (5.5)	0.019^†^
**Spinal deformity index: mean (SD)**	0.6 (1.1)	0.2 (0.8)	0.2 (1.1)	0.499

*AxSpA* axial spondyloarthritis, *TBS* trabecular bone score, *BASDAI* The Bath Ankylosing Spondylitis Disease Activity Index, *ASDAS* The Ankylosing Spondylitis Disease Activity Score.

*Statistically significant when comparing among the three groups (degraded bone group, partially degraded bone group, and normal bone group).

^†^Statistically significant when comparing the degraded bone group with the normal bone group.

^$^Included individuals with Z-score below -2.0.

^#^Not included 3 cases in whom the radiologist could not interpret VFs due to artifact.

^§^Statistically significant when comparing the degraded bone group with the partially degraded bone group.

^¶^Statistically significant when comparing the partially degraded bone group with the normal bone group.

### Factors associated with the trabecular bone score

In the univariate linear regression, ex- or current smoker, alcohol intake ≥ 3 units/day, BMI, current NSAIDs use, all parameters of disease activity, presence of syndesmophyte, and grade 4 sacroiliitis were associated with low TBS. In multivariate linear regression analysis, TBS was negatively associated with female gender, ex- or current smoker, alcohol intake ≥ 3 units/day, ASDAS, and grade 4 sacroiliitis, whereas BMI was positively associated with TBS.

The univariate logistic regression model showed that higher disease severity measurements including PGA, ESR, hsCRP, BASDAI, BASMI, BASFI, ASDAS, grade 4 sacroiliitis, and the presence of syndesmophyte, alcohol intake ≥ 3 units/day, shorter disease duration, and PPI uses were significantly associated with degraded bone/low TBS. The ASDAS and grade 4 sacroiliitis were respectively selected to represent current disease severity and damage in multivariate models. In multivariate logistic regression, ASDAS, and grade 4 sacroiliitis were independently associated with degraded bone/low TBS with the adjusted odds ratios (confidence interval: CI) of 5.228 (1.611–16.963) and 11.820 (1.211–115.412), respectively. Short disease duration was also found to be associated with degraded bone/low TBS; although the association was modest with an adjusted odds ratio (CI) of 0.982 (0.966–0.998), (Table 3).

**Table 3:** Linear regression for TBS and logistic regression for degraded bone/low TBS in patients with axSpA.

Variable	Linear regression		Logistic regression (n = 131)	
	Univariate model, β (95%CI)	Multivariate model^†^ (n = 127), β (95%CI)	Univariate model, OR (95% CI)	Multivariate model, adjusted OR^‡^ (95%CI)
Demographic				
Age, years	0 (−0.002, 0.001)		0.962 (0.905–1.024)	
Gender, female	−0.028 (−0.070, 0.014) ^#^	−0.052 (−0.093, −0.011)*	2.23 (0.589–8.443)	
Ex- or current smoker	−0.042 (−0.080, −0.005) *	−0.054 (−0.090, −0.017)*	1.200 (0.322–4.477)	
Alcohol intake ≥ 3 units/day	−0.057 (−0.102, −0.013)*	−0.059 (−0.102, −0.016)*	2.986 (0.779–11.441)^#^	
BMI, kg/m^2^	0.005 (0.001, 0.009)*	0.006 (0.002, 0.010)*	0.971 (0.835–1.128)	
Duration of disease	−0.000023(−0.000 to 0.000)		0.986 (0.971–1.002)^#^	0.982 (0.966–0.998)
HLA B27-positive	0.008 (−0.056, 0.072)		0.506 (0.051–4.990)	
Medication				
Current NSAIDs use	−0.058 (−0.093, −0.023) *		–^§^	
Current PPIs use	−0.028 (−0.065, 0.010)^#^		2.795 (0.749–10.434)^#^	
Current SSZ use	−0.001 (−0.037, 0.036)		0.750 (0.207–2.721)	
Current IL-17 inhibitor	−0.112 (−0.262, 0.037)		–^§^	
Current TNF inhibitor	0.001 (−0.047, 0.048)		–^§^	
Disease severity assessment				
PGA	−0.001 (−0.002, 0.000)*		1.037 (1.010–1.064)*	
ESR, mm/h	−0.001 (−0.002, 0.000)*		1.024 (0.999–1.050)^#^	
CRP, mg/l	−0.001 (−0.002, 0.000)*		1.028 (1.009–1.047)*	
BASDAI	−0.012 (−0.021, −0.004)*		1.485 (1.113–1.982)*	
BASMI	−0.015 (−0.023, −0.007)*		1.326 (1.008–1.744)*	
BASFI	−0.010 (−0.018, −0.003)*		1.234 (0.981–1.552)^#^	
ASDAS	−0.035 (−0.050, −0.020)*	−0.025 (−0.040, −0.010)*	5.018 (2.145–11.737)*	5.228 (1.611–16.963)
Grade 4 sacroiliitis	−0.064 (−0.100, −0.029)*	−0.046 (−0.082, −0.011)*	16.568 (2.032–135.074)*	11.820 (1.211–115.412)
Syndesmophyte	−0.054 (−0.090, −0.018)*		3.086 (0.629–15.126)^#^	
Adjusted R^2^		0.297	Nagelkerke R square	0.574

*p < 0.05.

^#^p < 0.20.

^†^Adjusted for gender, ex- or current smoker, alcohol intake ≥ 3 units/day, BMI, current NSADIs use, current PPIs use, Hb, BASDAI, BASMI, BASFI, ASDAS, grade 4 sacroiliitis, presence of syndesmophyte.

^‡^Adjusted for alcohol intake ≥ 3 units/day, duration of disease, current PPIs use, BASDAI, BASMI, BASFI, ASDAS, grade 4 sacroiliitis, syndesmophyte.

^§^Statistical analysis cannot be conducted due to a zero value within one of the TBS groups.

**Figure 1:**
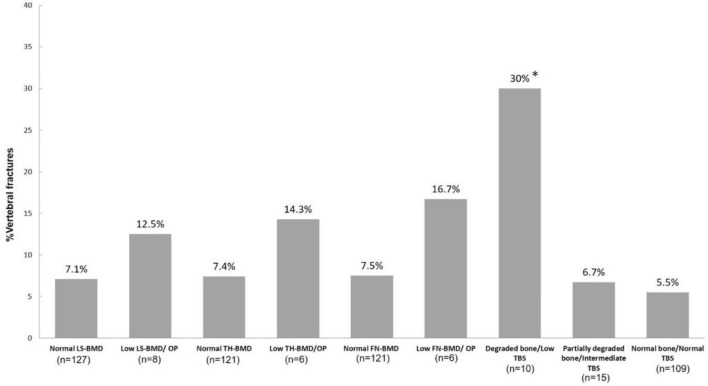
The prevalence of vertebral fractures (two cases were not included in which the radiologist could not interpret vertebral fractures due to artifacts) (VFs) stratified by low bone mineral density (BMD) or osteoporosis (OP) at the lumbar spine (LS), total hip (TH), femoral neck (FN), and trabecular bone score (one patient, whose TBS data could not be analysed due to morbid obesity, was excluded) (TBS) risks in patients with axial spondyloarthritis (axSpA). *p < 0.05, compared with normal TBS group.

VFs were found in ten patients in this study (7.5%). The prevalence of VFs was significantly higher in the degraded bone/low TBS group than in the intermediate and normal TBS groups (30.0% vs 6.7% vs 5.5%, p = 0.019, respectively), (Fig. 1). For identifying patients with VFs, the sensitivity (95%CI) and specificity (95%CI) of degraded bone/low TBS were 30% (6.7% to 65.3%) and 94.4% (88.7% to 97.7%), respectively, whereas the sensitivity (95%CI) and specificity (95%CI) of osteoporosis/low bone mass at LS-BMD and FN-BMD were 10.0% (0.25%-44.5%) and 94.4% (88.8%-97.7%) and 16.67% (0.42%-64.12%) and 92.56% (86.35%-96.54%), respectively.

**Figure 2:**
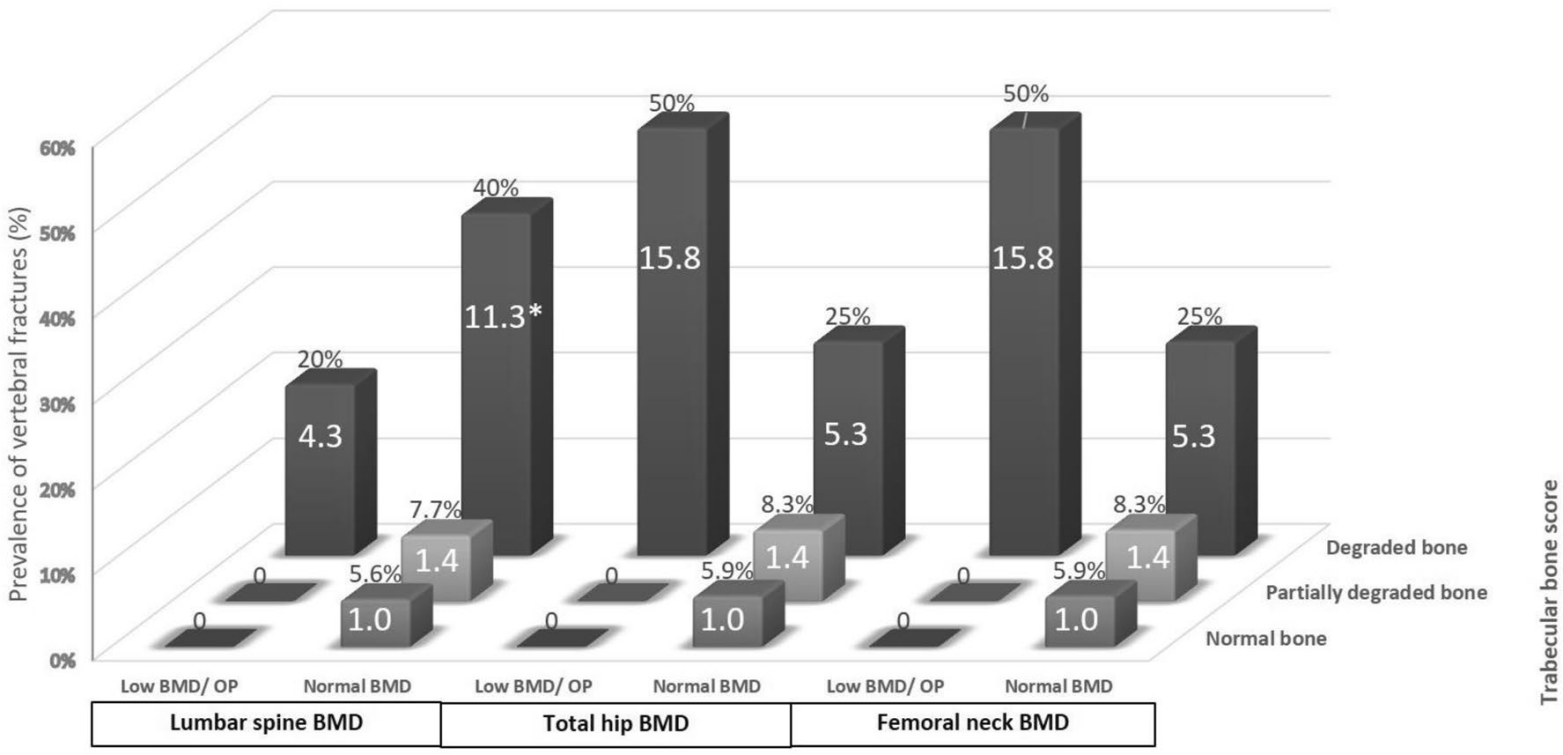
The prevalence of vertebral fractures, stratified by trabecular bone score (TBS) and osteoporosis/low bone mineral density (BMD) according to DXA. Asterisk: statistically significant when compared with patients with ax-SpA with normal BMD and normal TBS. The values displayed in the center of each bar represent the odds ratios for vertebral fractures when compared to individuals with both normal TBS and normal BMD at the lumbar spine, total hip, and femoral neck in patients with axSpA.

Combining the results from TBS and BMD, axSpA patients who had both degraded bone/low TBS and osteoporosis/low bone mass at the femoral neck or total hip were at the highest risk of having VFs as compared to patients with normal TBS and FN-BMD group with the OR (95%CI) of 15.8 (0.88–285.48), p = 0.061 for both femoral and total hip site. In contrast, axSpA patients that had normal BMD and degraded bone/low TBS at the lumbar spine had an increased risk of VFs as compared with those with normal BMD and normal TBS at the same site with the OR (95%CI) of 11.3 (1.58–81.23), p = 0.016), (Fig. 2).

## Discussion

In this study, patients with axSpA had significantly lower TBS values than sex-age-matched healthy volunteers. The prevalence of degraded bone/low TBS was higher in axSpA patients when compared with healthy volunteers. VFs were more common in patients in the degraded bone/low TBS group than in the intermediate and normal groups. Female gender, smoking status, alcohol intake greater than or equal to 3 units/day, shorter disease duration, elevated hs-CRP, higher disease activities, current NSAIDs use, and not receiving anti-TNF or anti-OP drugs were all significantly associated with lower TBS in patients with axSpA.

The findings from this study were consistent with previous studies^25–28^. High disease activity related to inflammation and new bone formation, impaired function, and spinal mobility as assessed by BASFI and BASMI were found to be associated with low TBS in axSpA patients. These findings were supported by an MRI study of the spine, which revealed that local inflammation, known as bone marrow edema, was detected in trabecular bone regions^29^ as well as the presence of new bone formation on plain films, such as fusion of sacroiliac joints and syndesmophytes, were associated with low TBS in axSpA patients^30–32^.

A meta-analysis of seven longitudinal studies and one randomised controlled trial reported that anti-TNF treatment increased LS-BMD in AS patients by 5.1% and 8.6% after 1 and 2 years, respectively^33^. Another prospective study in 12 AS patients found that, despite a significant increase in LS-BMD, there was no significant improvement in TBS after 2 years of anti-TNF treatment^34^. This study found that axSpA patients who did not receive anti-TNF treatment had lower TBS than those who did and that low TBS was associated with high disease activity. These findings emphasized the significance of controlling disease activity with anti-TNF agents or other agents, as they could improve bone quality (TBS) and bone mass (LS-BMD) in axSpA patients.

In this study, alcohol and smoking were associated with degraded bone/low TBS in axSpA patients. Several studies in postmenopausal women found that alcohol consumption and smoking were strongly associated with low BMD^33,35^; although, these associations had not previously been reported in axSpA patients^27,28^. Furthermore, those risk factors have been linked to increased radiographic progression in patients with axSpA^36,37^. As a result, physicians should encourage axSpA patients to discontinue the modified risk factors.

This study found that shorter disease duration was independently associated with degraded bone/low TBS; although, the association was modest. This finding supported the Danish Health Registries’ findings that patients with AS had an increased risk of clinical vertebral and non-vertebral fractures, with the highest risk occurring within the first 2.5 years after diagnosis^38^. In addition, previous studies demonstrated that a decrease in LS-BMD was associated with active inflammatory lesions at the lumbar spine and sacroiliac joints in early AS patients^39,40^, and the 2-year follow-up of the DESIR cohort also showed that the prevalence of low BMD at the lumbar spine was 22.4% in patients with early axSpA^41^. Patients with a briefer disease duration may experience more severe symptoms and disease progression, leading them to seek medical attention at an earlier stage. The appropriate bone health assessment should be performed as part of an initial treatment plan in axSpA patients.

AxSpA patients in the degraded/low TBS groups had a higher prevalence of VFs and a higher risk of developing new VFs when compared to those in the normal or intermediate TBS groups^27,28,42–44^. Degraded bone/low TBS had higher sensitivity than FN-BMD and LS-BMD for detecting VFs in axSpA patients without losing specificity. Although the 2019 ACR recommendations suggested patients with axSpA should be screened for osteoporosis with a DXA scan of the spine and hip^10^, the results of previous studies together with findings from this study suggested TBS provided additional benefit for vertebral fracture assessment than LS-BMD and FN-BMD alone since vertebral fractures in axSpA patients from this study occurred at a normal BMD.

The clinical application of TBS for fracture risk assessment in patients with axSpA is not well established. TBS could be used in two ways to assess fracture risk in axSpA patients. First, degraded bone/low TBS level could be a tool for fracture risk assessment in AS patients. The cut-off for degraded bone/low TBS used in this study was derived from a meta-analysis of 14 prospective population-based cohorts from countries around the world examining TBS in fracture risk prediction^19^. However, it is unclear whether this threshold could be used in axSpA patients of any ethnicity since ethnicity affects the fracture-predictive ability of TBS^45^. Secondly, the value of TBS could be incorporated in FRAX (fracture assessment tool) along with clinical risk factors and FN-BMD. Further research into the clinical use of FRAX and TBS is needed to determine the appropriate adjustment to assess fracture risk in axSpA patients.

There were some limitations in this study. First, this study did not examine some other risk factors for osteoporosis such as vitamin D level, serum calcium, and iPTH. Second, there were limited numbers of patients with low TBS and VFs and only a few patients had other major osteoporotic fractures. Third, as a cross-sectional study, this study data might not represent overall disease activities in patients with axSpA, although the results showed that all parameters of disease activity were consistent with the severity of the disease. Furthermore, only a few patients were menopause or had already received anti-osteoporotic treatment; although, after excluding post-menopausal status or anti-osteoporotic treatment, the results were indifferent.

## Conclusion

Patients with axSpA have lower TBS than healthy volunteers. The proportion of axSpA patients with degraded bone/low TBS was higher than the proportion of healthy volunteers. TBS was found to be independently associated with gender, former or current smoker, ≥ 3 units/day alcohol consumption, low BMI, high disease activity including PGA, BASDAI, BASFI, BASMI, and ASDAS, and fusion of sacroiliac joints. Therefore, TBS is a good non-invasive tool for assessing fracture risk in axSpA patients. TBS can be used alone or in conjunction with BMD measurement to identify axSpA patients at high risk for vertebral fractures.

## Data Availability

The data that support the findings of this study are available at reasonable request from the corresponding author.

## Abbreviations

axSpA: Axial spondyloarthritis
SI: Sacroiliac
IL: Interleukin
TNF-α: Tumor necrosis factor-α
BMD: Bone mineral density
VFs: Vertebral fractures
CRP: C-reactive protein
DXA: Dual-energy X-ray absorptiometry
ACR: The American College of Rheumatology
LS: Lumbar spine
FN: Femoral neck
TH: Total hip
VFA: Vertebral fracture assessment
BMI: Body mass index
NSAIDs: Non-steroidal anti-inflammation drugs
PPIs: Proton pump inhibitors
PGA: Patient global assessment
BASDAI: The Bath Ankylosing Spondylitis Disease Activity Index
ASDAS: The Ankylosing Spondylitis Disease Activity Score
BASFI: The Bath Ankylosing Spondylitis Functional Index
BASMI: The Bath Ankylosing Spondylitis Metrology Index
CI: Confidence interval
